# Improved method of fire hazard assessment taking into account the effect of the primary temperature of a coal seam on the desorption ratio of gas indicators

**DOI:** 10.1038/s41598-022-09695-8

**Published:** 2022-04-05

**Authors:** Marek Więckowski, Natalia Howaniec, Adam Smoliński

**Affiliations:** 1 Department of Mining Aerology, Central Mining Institute, Plac Gwarków 1, 40-166 Katowice, Poland; 2 Department of Energy Saving and Air Protection, Central Mining Institute, Plac Gwarków 1, 40-166 Katowice, Poland; 3 Central Mining Institute, Plac Gwarków 1, 40-166 Katowice, Poland

**Keywords:** Environmental sciences, Environmental social sciences, Natural hazards, Engineering

## Abstract

The methods of fire risk assessment in its early phase employed so far used to take into account only the temperature of the heating of coal. The research works reported so far in this field have been conducted at ambient temperatures, without considering the rock mass primary temperature occurring in a particular coal seam and neglecting the depth of coal seams. The method for the determination of the composition of gases emitted from coal samples, employed in the study presented here, takes into account the effect of rock mass pressure, and therefore the depth of the coal seam, as well as the temperature typical for the rock mass in a particular coal mine. In the paper the results of the experimental research on the effects of the primary temperature of a coal seam on the content of gases emitted from coal, performed with the use of a specially designed experimental stand are presented. The method may be useful in developing fire hazard predictions for longwalls with residual coal in goafs. The relations between the self-ignition characteristics as well as selected physical properties of coal samples and carbon monoxide emission were also given. The research results proved that the accurate fire hazard assessment requires considering other than just self-heating of coal causes of carbon monoxide emission, including the rock mass primary temperature, which is expected to improve the existing methods of fire risk assessment in coal mines and post-mining areas.

## Introduction

Exogenous fires pose a severe challenge in bituminous coal extraction practice. In recent years, research studies concerning the process of coal self-heating and the development of new methods dedicated to the assessment of this phenomena have been carried out in many countries^1–4^. The fire hazard assessment in bituminous coal mining industry nowadays employs the measurements of concentration of gases released in the process of coal oxidation, including carbon monoxide, ethylene, propylene, acetylene and hydrogen^1,5,6^. Such methodological approach has been in use for decades and is based on the observation that the contents of the above mentioned gaseous indicators in a mine atmosphere increases with the temperature of coal self-heating. In particular, rapid increases in the contents of carbon monoxide and hydrogen are observed above the temperature of coal ignition.

The phenomena of coal self-heating are accompanied by numerous processes hindering the appropriate assessment of fire hazard, including the natural desorption of gases from coal^7,8^. The main gas emitted during the process of low temperature self-heating of coal is methane, while carbon monoxide was proven and implemented as the main indicator of the development of low temperature self-ignition of coal^9^. The variations in particular gaseous components concentrations in a mine air may be also independent from coal self-heating, which was demonstrated by Singh et al.^10^.

Nevertheless, with the temperature increase, the proportions between the concentrations of particular gaseous components change, creating a specific atmosphere background for a given coal seam. Coal oxidation, under the low temperature conditions, is considered to be the predominant source of heat provided to the process of self-heating of coal in underground mines and the main gas emission driver^11–13^.

In the attempts of the description of the oxidation process mechanism reported in the literature various parameters are applied in the development of kinetic models, covering coal sample weight loss, heat release, oxygen demand, as well as kinds and amounts of gaseous and solid products^14^. The effect of an air inflow to coal beds is also studied as one of the factors facilitating the development of fires in underground mines^15,16^.

When it comes to analyzing in details the sources of carbon monoxide emission it has already been reported that the two main are: coal crushing and oxidation at ambient temperature^17^. It has also been proven in studies on the impact of moisture, primary temperature, as well as the content of seam gas and reactive pyrite on coal self-heating rates at low temperatures using adiabatic tests, that gases characteristic for a mine atmosphere may be also emitted as a result of natural desorption^18^.

In the study on gas release in the process of coal production higher initial amounts and rates of gas desorption were observed for fragmented coal than for the body of coal^19^. Carbon monoxide is also naturally present within the seams of coal (irrespective of coal self-ignition process) and is detected during the course of mining activities at the levels of up to several hundred ppm^20,21^. Fracturing of coal in the process of coal extraction may also itself release gases from coal structure with the values of carbon monoxide concentrations depending on the coal rank, the mode and depths of the exploitation as well as the type of the overlying rocks. Pressure and temperature also have an influence on coal sorption properties^22–25^.

Therefore, it should be taken into account that the simply mechanical factors may also affect (increase) the values of gas concentrations in a coal mine atmosphere and consequently the correctness of the assessment of coal self-heating status, performed on the basis of carbon monoxide concentrations detected in a mine atmosphere and determining the specified, legally required fire-fighting activities^26^.

In underground mining, the zone of full roof failure includes roof strata of the thickness equal to 1–2 times the thicknesses of the seam. Peng^27^ gave the principles of the calculation of the vertical roof failure with respect to rock relaxation coefficient. The increasing amount of rocks fills the post-extraction space, which finally meets the roof strata with no transformation into the roof failure state. The rock debris is bent and squeezed and in consequence cracked and delaminated by the roof strata over the full failure zone, creating the high roof failure zone of the height of 1–1.5 times of the seam thickness. The full and the high roof failure zones form the roof strata of the thickness of 3–3.5 times of the seam thickness, which enables estimation of the force effect of the roof strata on the residual coal. Thus, intensified extraction of coal creates conditions facilitating desorption of carbon monoxide from longer walls, by affecting the range of the stress relief in the surrounding bed, and therefore also the volume of the degassed bed. This makes the applicability of carbon dioxide as a coal self-heating indicator arguable^28^, while the enhanced release of carbon monoxide from the body of coal is considered to be useful in these terms^29^.

Crushing of coal, taking place in the goafs of longwall increases the surface area of coal bed exposed and thus intensifies gas desorption. The depth of coal extraction affects the primary temperature of the rock mass (e.g. in Polish coal mines it varies from approximately 20 to 50 °C), and therefore the rise in carbon monoxide content in a mine atmosphere cannot be considered solely as a certain prove of coal self-heating.

In the light of the above, a new, more precise method of fire hazards assessment, based on the analysis of carbon monoxide concentrations related both to the primary temperature of the rock mass and the depth of a seam is needed, which was also proven experimentally in the study presented in this paper on the basis of the research results acquired with the use of the unique experimental set-up. This test stand was designed to enable simulation of the coal crushing process behind the roof support and under the conditions of the primary temperatures in the workings. Thus, the innovativeness of the approach adopted in the study presented covers two main methodological aspects: taking into account the effect of rock mass pressure (depth of the coal seam) and the temperature, typical for the rock mass in a particular coal mine, and the research installation specially designed and constructed, and applicable in simulation of the process of coal crushing behind the roof support, and under the conditions of the primary temperatures in the workings. The research results presented in the paper are therefore considered to be contributing to the improvement of the fire hazard assessment in coal mines.

## Materials and methods

### Materials

In order to determine the amount of carbon monoxide emitted at primary temperature of the rock mass, coal was sampled from a freshly uncovered longwalls, at the largest possible mass, and transported to the laboratory in metal, airtight vessels. The physical and chemical characteristics of coal samples are given in Table 1.

**Table 1:** The physical and chemical characteristics of coal samples.

Sample no.	Seam	Transient moisture content (%)	Analytical moisture (%)	Ash (%)	Self-ignition index, Sz^a^ (^o^C/min)	Self-ignition group	Dry ash free coal content, z_daf_ (%)	Dry ash free coal mass, m_daf_ (g)	Rock mass primary temperature (°C )
1	416	0.7	1.2	4.5	73	II	93.6	1123.2	35
2	414	0.2	1.1	5.4	70	II	93.3	1119.6	34
3	414/2	0.4	1.6	1.6	68	II	96.4	1156.8	29
4	212	6.2	12.9	8.3	111	IV	72.6	871.2	23
5	215	2.5	5.6	10.1	114	IV	81.8	981.6	22
6	408	0.3	2.7	2.2	90	III	94.8	1137.6	26
7	501	1.1	1.7	4.5	86	III	92.7	1112.4	27
8	404	0.6	0.6	5.5	49	I	93.3	1119.6	29
9	206	10.1	6.9	4.6	128	V	78.4	940.8	22
10	330	1.2	3.5	6.7	88	III	88.6	1063.2	27
11	408	1.7	1.6	5.5	75	II	91.2	1094.4	36
12	409	0.3	1.2	3.2	50	I	95.3	1143.6	41
13	505	1.7	1.1	6.1	42	I	91.1	1093.2	43
14	405	1.8	1.1	4.6	63	II	92.5	1110.0	48
15	207	6.5	8.8	5.6	131	V	79.1	949.2	21

### Methods

The research method consists in degassing the samples of crushed coal at four temperatures. For the purpose of the study presented in this paper, the following temperatures were predetermined: 20, 30, 40 and 50 °C, reflecting the primary temperature ranges occurring in Polish mines.

**Figure 1:**
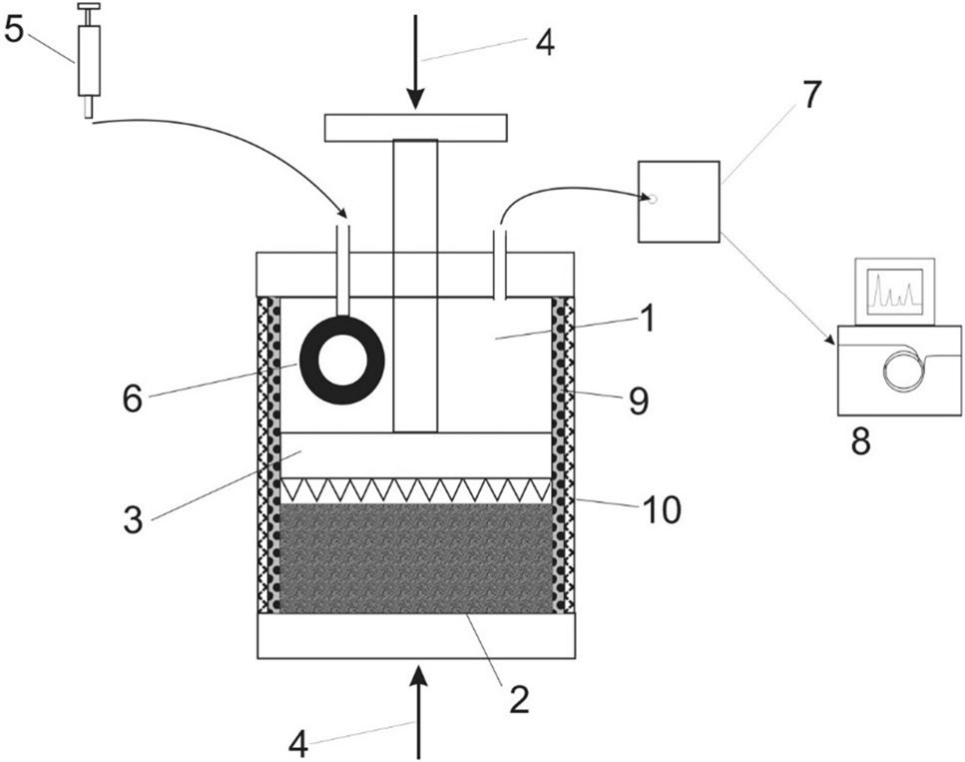
The schematic of the test stand for studying the natural emission of gases. 1—hermetic vessel, 2—sample, 3—crushing punch, 4—press, 5—pump, 6—rubber balloon, 7—Tedlar bag, 8—GC, 9—heating mat, 10—thermal insulation.

The crushing of coal takes place inside a research vessel without external air access. In the course of the experiment, the concentration of carbon monoxide in gaseous samples is determined. The test stand is presented in Fig. 1. The main element of the installation is a hermetic vessel into which samples of 1200 g of coal in grain size 10–30 mm are put, tightly locked and crushed with a crushing punch and the pressing force of 20 MPa. Next the desorption of gasses to the free space of the vessel is allowed to proceed for 24 h and then the tested gaseous mixture is driven away from the vessel by inflating a rubber balloon inside it to avoid diluting the tested mixture with atmospheric air. Therefore, the design of the device allows for the natural crushing of coal, taking into account the pressure of the rock mass. The second important feature of the device is its capability to simulate the primary temperature of the rock mass. A large mass of coal allows eliminating the inhomogeneity occurring in coal seams. These features makes the experimental conditions adopted better reflect the the conditions of a coal mine.

Shimadzu GC 2014 gas chromatograph (GC) with a TCD detector, a molecular sieve column and a Porapak column for determination of concentrations of O_2_, N_2_, CH_4_, CO and CO_2_ is employed.

The content of dry and ash free coal in a sample, *z*_*csw*_, is calculated as^30,31^:

1 $$z_{csw} = 100 - \left( {W_{c} + A_{a} } \right),\;\% {\text{w}}/{\text{w}},$$

where *W*_*c*_—total moisture content, % w/w, *A*_*a*_—ash content, % w/w.

Then the mass of dry ash free coal, *m*_*csw*,_ is:

2 $${m}_{csw}=\frac{{m}_{w \cdot }{z}_{csw}}{100}, \mathrm{g},$$

where *m*_*w*_—coal sample mass, g.

The volumes of coal sample, *V*_*w*_, and free space in the vessel are 1533 cm^3^ and 10,350 cm^3^, respectively.

The volume of CO in tested gaseous mixture, *V*_*CO*_, is calculated as:

3 $${V}_{CO}=\frac{{{V}_{o}\cdot S}_{CO}}{100}, {\mathrm{cm}}^{3},$$

where *V*_0_—degassed mixture volume, cm^3^, *S*_*co*_—carbon monoxide content in degassed mixture, % v/v.

Carbon monoxide content in a sample is:

4 $$\mathrm{CO}=\frac{{V}_{CO}}{{m}_{csw}}, {\mathrm{m}}^{3}\mathrm{CO}/{\mathrm{Mg}}_{\mathrm{csw}}.$$

The self-ignition index^32^ is also determined.

## Results and discussion

**Figure 2:**
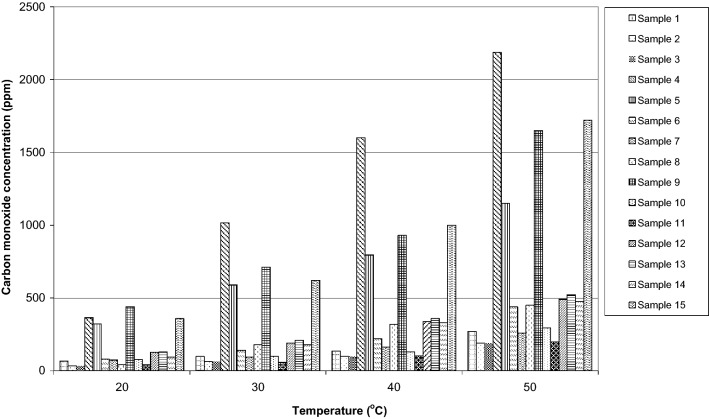
Contents of carbon monoxide in gas released from coal at 20, 30, 40 and 50 °C.

The analyses of selected gaseous components release were conducted for 15 bituminous coal samples exposed to 20 MPa, simulating the rock mass pressure at the depth of 800 m and at the temperature of 20, 30, 40 and 50 °C, simulating the rock mass primary temperature in the studied coal seams. The obtained results demonstrated that carbon monoxide desorption increases rapidly with the rise in the rock mass primary temperature and the amount of carbon dioxide released varies for various samples (Fig. 2).

**Figure 3:**
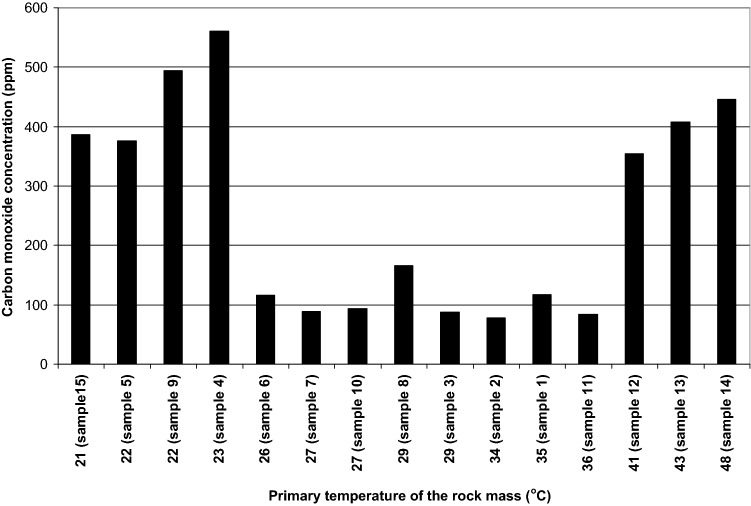
Contents of carbon monoxide in gas released from coal vs rock mass primary temperature.

Figure 3 presents these values arranged according to the increasing rock mass primary temperature.

In general, the contents of carbon monoxide at low primary temperatures (26–36 °C) were low and increased above 41 °C. The exception was reported for samples 4, 5, 9 and 15, which showed high concentrations of carbon monoxide despite the fact that the rock mass temperature occurring in these seams was low and ranged from 21 to 23 °C.

**Figure 4:**
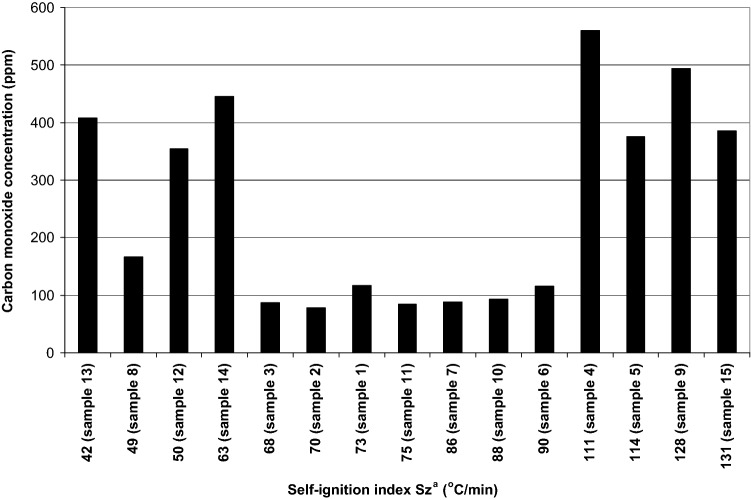
Contents of carbon monoxide in gas released from coal vs self-ignition index.

The correlation between the content of carbon monoxide desorbed from a coal sample and the respective moisture content and the self-ignition index value was also observed (Fig. 4).

As it can be seen from Fig. 4, significant amounts of carbon monoxide were desorbed from previously mentioned samples 4, 5, 9 and 15, characterized by the highest values of the self-ignition index (self-ignition groups IV and V) (Table 1). Interestingly, samples 12, 13 and 14, of relatively low values of the self-ignition index also demonstrated high concentrations of carbon monoxide. In Table 2 the amounts of carbon monoxide recalculated to mass dry ash free coal substance are presented.

**Table 2:** The content of carbon monoxide recalculated to mass of dry ash free coal substance.

Sample no.	Carbon monoxide concentration (ppm)	Carbon monoxide content (m^3^_CO_/Mg_csw_)
1	117	0.001042
2	78	0.000700
3	88	0.000758
4	561	0.006435
5	376	0.003826
6	116	0.001020
7	88	0.000795
8	166	0.001484
9	495	0.005257
10	94	0.000881
11	84	0.000771
12	355	0.003104
13	408	0.003732
14	446	0.004018
15	386	0.004067

The values of the total moisture content in coal samples tested (Table 1) varied from 1.2%w/w for sample 8 to 19.1%w/w for sample 4. It has been previously reported that the sorption capacity of coal may decrease with high ash content^33^. In the study presented in this paper, however, the ash content was low, in the range from 1.6%w/w (sample 3) to 10.1%w/w (sample 5) and therefore no direct impact of this parameter on carbon monoxide desorption was observed.

**Figure 5:**
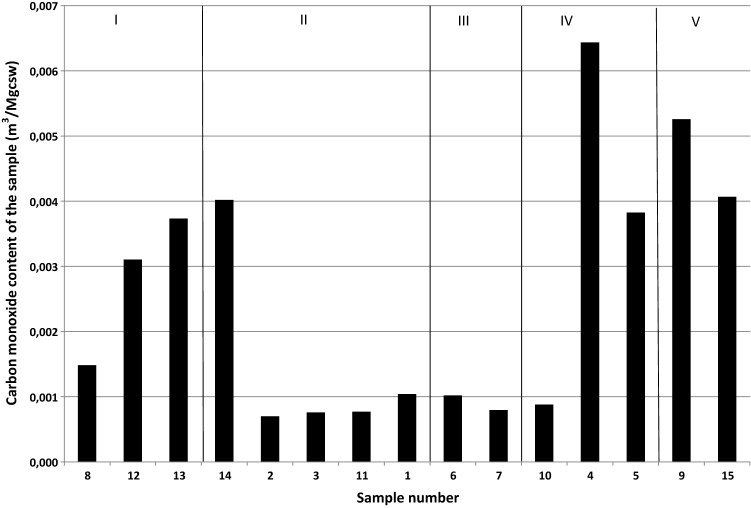
Contents of carbon monoxide in gas released from coal per mass of dry ash free coal substance.

The oxidation heat depends on the mass of the coal which undergoes the oxidation process. The content of dry ash free coal substance in the examined samples, z_csw_, was from 72.6%w/w for sample 4 to 96.4%w/w for sample 3 (Table 1). Figure 5 shows volumes of carbon monoxide released for temperature conditions characteristic of particular seams and recalculated to the mass of dry ash free coal substance.

The higher the self-ignition group, the more intensive the emission of carbon monoxide was still observed after the above recalculation, and taking into account the contents of ash and moisture in coal samples tested. Another correlation, not related to the coal self-ignition group, but to the rock mass primary temperature, was also reported for samples 12, 13 and 14.

Research studies on gas adsorption/desorption on/from bituminous coals reported in the literature most commonly refer to the combination of carbon dioxide and methane^34^. Based on the knowledge on the structure of bituminous coals, and especially on the nature of coal surface as well as the studies on sorption processes, the authors presented interpretations of the research results with reference to the changes occurring during coal self-heating. The conditions of the experiments adopted in these studies, however, poorly reproduce the conditions of a coal mine, to mention only the mass and granulation of coal and vacuum degassing of the samples.

Natural desorption of gases from bituminous coal results in methane release to the atmosphere with smaller amounts of ethane, propane and carbon dioxide^35–37^. Extensive studies in this field were performed by Xu et al.^38,39^ and Chen et al.^40^. Exposing of coal to air results in parallel processes of coal oxidation and desorption of carbon monoxide and dioxide^41^. The product of early coal oxidation, detected in the highest concentrations and at low temperatures, both under the underground or laboratory conditions is carbon monoxide. Trace amounts of other gases make them difficult to be detected at this stage^42^. In case of natural desorption, the rise in carbon monoxide content takes place. Fan et al.^43^ on the basis of the experimental study on the sorption of the primary carbon monoxide in coal seams concluded that its adsorption mechanism is similar to the adsorption of methane. The applicability of the method originally dedicated to measuring the amount of methane, also to carbon monoxide, was demonstrated. The results of the study also confirmed that the primary carbon monoxide is present in Xichuan colliery, and that the amount of this gas changes with the location, reaching the value of 487.79 × 10^–6^ m^3^_CO_/Mg and is not related to the process of coal self-heating. Three explanations for the occurrence of carbon monoxide were given: (i) release in coal extraction, (ii) release from rock strata cracks caused by coal extraction, and (iii) oxidation of residual coal^43^.

Zhang et al.^44^ measured the carbon monoxide emission under non-isothermal conditions using an apparatus of their own design and construction, and coal type, grain size and oxygen content in coal as variables adopted in the experimental procedure. They found that the application of coal of larger particle diameters resulted in decreasing the emission of carbon monoxide, particularly at higher temperatures.

Xu et al.^45^ also studied the process of coal self-heating at low temperatures. In their experiment, the concentrations of gaseous products as well as the temperature inside the research vessel were measured when the coal samples were subjected to heating. Based on data analysis, four stages of the oxidation process were identified: (i) preliminary heating without oxidation reaction or volatilization, (ii) volatilization without oxidation, (iii) unevenly accelerated oxidation with parallel vaporization, and (iv) late heating stage, when the entire volume of oxygen is consumed in the oxidation reaction. Tang^17^ also confirmed the correlation between the grain size of coal and carbon monoxide release intensity: the finer the coal sample was ground (1–3 mm) with a ball grinder, the higher the amount of carbon monoxide was produced. The concentration of carbon monoxide released was also lower for coal samples of higher moisture content.

Xie et al.^46^ found that the presence of certain oxidation products may facilitate identification of particular phases and the intensity of the coal self-heating process. The sequence in which the products occur varied depending on coal types. However, the mechanism of these phenomena, and in particularly the effects of various factors on the initiation and the development of the self-heating process, has been reported to be still unrecognized.

In the research works presented in this paper the share of grains above 0.7 mm constituted above 70% of the coal sample; therefore, the analyses performed with porosimeters would not reflect the coal mine conditions. The experimental stand employed in the study allowed desorbed gas sampling at ambient pressure and with no air–derived diluting effect.

**Figure 6:**
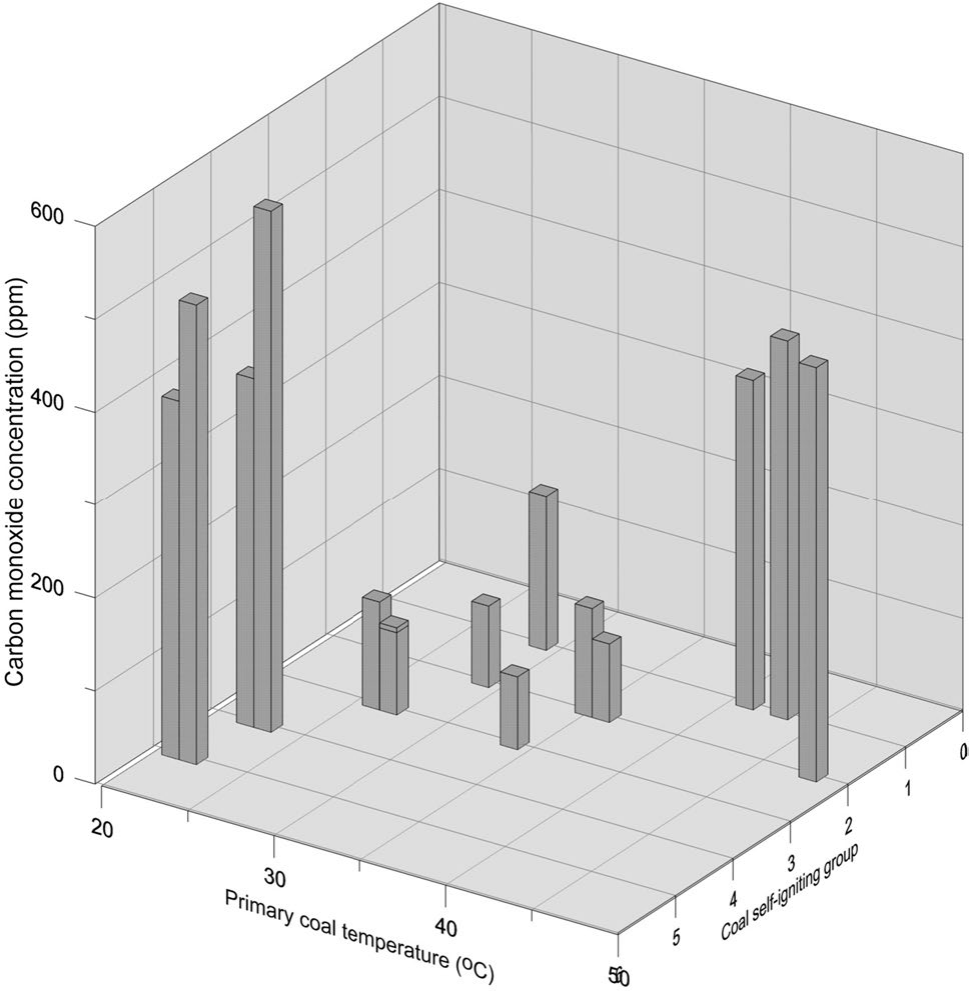
Contents of carbon monoxide in gas released from coal vs the primary temperature and coal self-ignition group.

On the basis of the research results presented in this paper, it may be concluded that coal samples of the highest moisture content (samples 4, 5, 9 and 15, Table 1) released the highest amounts of carbon monoxide. However, a multi-dimensional approach is needed to accurately estimate the emissions of carbon monoxide and other gases; the coal self-ignition group or self-ignition index, as well as the rock mass primary temperature occurring at the given seam depth have also to be taken into account. A complex analysis of these dependencies is presented in Fig. 6.

As it has been previously mentioned the reported amounts of carbon monoxide desorbed were high for samples originating from high coal self–ignition groups (IV and V): samples 4, 5, 9 and 15, acquired respectively from seams 212, 215, 206 and 207, at shallow depths, where the rock mass primary temperature only slightly exceeded 20 °C.

Another strong correlation was observed for the primary temperature: samples 12, 13 and 14, although of I and II self-ignition groups, also showed high amounts of carbon monoxide desorbed, which resulted from relatively high rock mass primary temperature values, of 41, 43 and 48 °C, respectively. Under such primary temperatures, coals theoretically not very prone to self–ignition, when taking into account their self-ignition groups, release under the mine conditions the amounts of carbon monoxide comparable to coals particularly prone to the self-ignition process.

To sum up, on the basis of the experimental results presented in this paper, the coal samples studied may be divided into the following groups:

- Coals emitting less than 0.001 m^3^_CO_/Mg_csw_; with the rock mass primary temperature below 40 °C and of the self-ignition groups of II and III (samples 1, 2, 3, 6, 7, 10 and 11).
- Coals emitting over 0.001 m^3^_CO_/Mg_csw_; with the rock mass primary temperature above 40 °C and of the self-ignition groups of I and II (samples 12, 13 and 14).
- Coals emitting over 0.003 m^3^_CO_/Mg_csw_; with the rock mass primary temperature below 25 °C and of the self-ignition groups of IV and V (samples 4, 5, 9 and 15).

The primary temperature of the rock mass surrounding the longwall affects significantly the amounts of carbon monoxide released and therefore testing of coal at laboratory temperature leads to erroneous conclusions. The same coal at the laboratory temperature will emit much lower concentrations of carbon monoxide than at the primary temperature of the rock mass. The higher is the temperature of the rock mass, the greater the error in the correct assessment of fire risk is. Since the concentrations of carbon monoxide and other gases are commonly employed as the indicators of coal self-heating progress, the desorption of gases and the factors affecting its intensity need to be taken into account, especially when coal extraction takes place at increasingly deeper depths.

## Conclusions

On the basis of the research results presented in this paper the following conclusions may be drawn:

1. The following correlations between the amount of carbon monoxide released and the physical and chemical characteristics of coal as well as the rock mass primary temperature was observed:
  Coal samples 4, 5, 9 and 15, of the highest self-ignition groups (IV and V), were characterized by the highest rate of carbon monoxide desorption, despite the fact that they were acquired from shallow coal seams of relatively low rock mass temperature, of 21–23 °C.
  Coal samples 12, 13 and 14, of the lowest self-ignition groups (I and II), but acquired from deeper coal seams, of the rock mass temperature above 40 °C, also desorbed significant amounts of carbon monoxide.
  The remaining coal samples, of self-ignition groups II and III, and taken from coal seams of the moderate rock mass temperatures (below 40 °C), emitted considerably smaller amounts of carbon monoxide.
2. When the depth of the coal bed (and the rock mass primary temperature) is taken into account in the method of fire hazard assessment, then the previously employed scheme of coal classification changes: coals of low self-ignition groups, under the conditions of high rock mass primary temperature, emit carbon monoxide in the amounts similar to these characteristic for coals of high self-ignition groups.
3. The rise in the rock mass primary temperature results in intensified natural desorption of gases commonly applied as indicators of the coal self-heating process development. This, in particularly, may result in detection of carbon monoxide concentrations similar to the ones characteristic for coal self-heating.
4. The accurate fire hazard assessment requires considering other than self-heating of coal causes of carbon monoxide emission, including the rock mass primary temperature.
5. The methodological approach proposed in this paper is expected to improve the existing methods of fire risk assessment in coal mines and post-mining areas.

## Data Availability

All data generated or analyzed during this study are included in this published article.

## References

[1] Adamus A, Sancer J, Guranova P, Zubicek V (2011). An investigation of the factors associated with interpretation of mine atmosphere for spontaneous combustion in coal mines. Fuel Process. Technol..

[2] Baris K, Kizgut S, Didari V (2012). Low-temperature oxidation of some Turkish coals. Fuel.

[3] Taraba B, Pavelek Z (2014). Investigation of the spontaneous combustion susceptibility of coal using the pulse flow calorimetric method: 25 years of experience. Fuel.

[4] Tsai YT, Yang Y, Wang C, Shu CM, Deng J (2017). Comparison of the inhibition mechanisms of five types of inhibitors on spontaneous coal combustion. Int. J. Energy Res..

[5] Dudzińska A, Howaniec N, Smoliński A (2015). Experimental study on sorption and desorption of propylene on Polish hard coals. Energy Fuels.

[6] Li Q, Wang C, Deng J, Shu C (2019). Thermokinetic characteristics of coal spontaneous combustion based on thermogravimetric analysis. Fuel.

[7] Więckowski M, Howaniec N, Postnikov EB, Chorążewski M, Smoliński A (2018). Changes in the distribution of temperature in a coal deposit and the composition of gases emitted during its heating and cooling. Sustainability.

[8] Song Z, Kuenzer C (2014). Coal fires in China over the last decade: A comprehensive review. Int. J. Coal Geol..

[9] Lu P, Liao GX, Sun JH, Li PD (2004). Experimental research on index gas of the coal spontaneous combustion at low-temperature stage. J. Loss Prevent. Proc..

[10] Singh AK, Singh RVK, Singh MP, Chandra H, Shukla NK (2007). Mine fire gas indices and their application to Indian underground coal mine fires. Int. J. Coal Geol..

[11] Zubicek V, Adamus A (2013). Susceptibility of coal to spontaneous combustion verified by modified adiabatic method under conditions of Ostrava-Karvina Coalfield, Czech Republic. Fuel Process. Technol..

[12] Xia T (2015). Simulation of coal self-heating processes in underground methane-rich coal seams. Int. J. Coal Geol..

[13] Arisoy A, Beamish B (2015). Mutual effects of pyrite and moisture on coal self-heating rates and reaction rate data for pyrite oxidation. Fuel.

[14] Wang J (2018). Assessment of spontaneous combustion status of coal based on relationships between oxygen consumption and gaseous product emissions. Fuel Process. Technol..

[15] Yuan L, Smith AC (2011). CO and CO_2_ emissions from spontaneous heating of coal under different ventilation rates. Int. J. Coal Geol..

[16] Więckowski M, Howaniec N, Smoliński A (2020). Effect of flow rates of gases flowing through a coal bed during coal heating and cooling on concentrations of gases emitted and fire hazard assessment. Int. J. Coal Sci. Technol..

[17] Tang Y (2015). Sources of underground CO: Crushing and ambient temperature oxidation of coal. J. Loss Prev. Process. Ind..

[18] Beamish BB, Theiler J (2019). Coal spontaneous combustion: Examples of the self-heating incubation process. Int. J. Coal Geol..

[19] Lu S (2015). Gas desorption characteristics of the high-rank intact coal and fractured coal. Int. J. Min. Sci. Technol..

[20] Wei GD (2000). Coal seam associates with CO. Inner Mongolia Coal Econ..

[21] Więckowski M, Howaniec N, Smoliński A (2020). Natural desorption of carbon monoxide during the crushing of coal simulating natural rock mass pressure. Sci. Total Environ..

[22] Iwaszenko S, Howaniec N, Smoliński A (2019). Determination of random pore model parameters for underground coal gasification simulation. Energy.

[23] Howaniec N (2016). The effects of pressure on coal chars porous structure development. Fuel.

[24] Howaniec N (2019). Combined effect of pressure and carbon dioxide activation on porous structure of lignite chars. Materials.

[25] Smoliński A, Howaniec N (2017). Analysis of porous structure parameters of biomass chars versus bituminous coal and lignite carbonized at high pressure and temperature—A chemometric study. Energies.

[26] Ministry of Economy (2016). Regulation of the Minister of Economy of 23.11. 2016 on Work-Related Safety, Conducting Mining Operations and Specialist Firefighting Systems in Underground Coal Mines.

[27] Peng S (2006). Longwall Mining.

[28] Dudzińska A, Cygankiewicz J (2015). Analysis of adsorption tests of gases emitted in the coal self-heating process. Fuel Process. Technol..

[29] Wang H, Dlugogorski BZ, Kennedy EM (2002). Examination of CO_2_, CO and H_2_O formation during low-temperature oxidation of a bituminous coal. Energy Fuels.

[30] Polish Committee for Standardization (1980). PN-G-04511 Solid Fuels, Determination of Moisture Content.

[31] Polish Committee for Standardization (1998). PN-G-04560 Solid Fuels. Determination of Moisture, Volatiles and Ash Contents with Automatic Analyzer.

[32] Polish Committee for Standardization (1993). PN-G-04558 Bituminous Coal. Determination of Self-ignition Index.

[33] Faiz M, Saghafi A, Sherwood N, Wang L (2007). The influence of petrological properties and burial history on coal seam methane reservoir characterization, Sydney Basin, Australia. Int. J. Coal Geol..

[34] Yu H, Zhou L, Guo W, Cheng J, Hu Q (2008). Predictions of the adsorption equilibrium of methane/carbon dioxide binary gas on coals using Langmuir and ideal adsorbed solution theory under feed gas conditions. Int. J. Coal Geol..

[35] Jin H, Schimmelmann A, Mastalerz M, Pope J, Moore T (2010). Coalbed gas desorption in canisters: Consumption of trapped atmospheric oxygen and implications for measured gas quality. Int. J. Coal Geol..

[36] Packham R, Cinar Y, Moreby R (2011). Simulation of an enhanced gas recovery field trial for coal mine gas management. Int. J. Coal Geol..

[37] Jiang Y, Song X, Liu H, Cui Y (2015). Laboratory measurements of methane desorption on coal during acoustic stimulation. Int. J. Rock Mech. Min..

[38] Xu Q (2019). Low-temperature oxidation of free radicals and functional groups in coal during the extraction of coalbed methane. Fuel.

[39] Xu Q (2020). Optimum oxidation temperature of coal bed for methane desorption in the process of CBM extraction. Fuel.

[40] Chen Y (2019). Differences in desorption rate and composition of desorbed gases between under-formed and mylonitic coals in the Zhina Coalfield, Southwest China. Fuel.

[41] Wang H, Dlugogorski BZ, Kennedy EM (2003). Coal oxidation at low temperatures: Oxygen consumption, oxidation products, reaction mechanism and kinetic modeling. Prog. Energy Combust. Sci..

[42] Gurdal G (2015). The properties of Çan Basin coals (Çanakkale–Turkey): Spontaneous combustion and combustion by-products. Int. J. Coal Geol..

[43] Fan J (2015). Adsorption and desorption characteristics of primary CO in coal bearing strata. Electron. J. Geotech. Eng..

[44] Zhang Y, Shi X, Li Y, Liu Y (2017). Characteristics of carbon monoxide production and oxidation kinetics during the decaying process of coal spontaneous combustion. Can. J. Chem. Eng..

[45] Xu Y, Wang L, Tian N, Zhang J, Yu M (2017). Spontaneous combustion coal parameters for the crossing-point temperature (CPT) method in a temperature-programmed system (TPS). Fire Saf. J..

[46] Xie J, Xue S, Cheng W, Wang G (2011). Early detection of spontaneous combustion of coal in underground coal mines with development of an ethylene enriching system. Int. J. Coal Geol..

